# Recent Advances on Metal-Free, Visible-Light- Induced Catalysis for Assembling Nitrogen- and Oxygen-Based Heterocyclic Scaffolds

**DOI:** 10.3390/molecules24081533

**Published:** 2019-04-18

**Authors:** Robert Pawlowski, Filip Stanek, Maciej Stodulski

**Affiliations:** Institute of Organic Chemistry Polish Academy of Sciences, Kasprzaka 44/52, 01-224 Warsaw, Poland; robert.pawlowski@icho.edu.pl (R.P.); filip.stanek@icho.edu.pl (F.S.)

**Keywords:** photocatalysis, photoredox, visible-light-induced catalysis, photoredox cyclization, organic dyes, heterocycles

## Abstract

Heterocycles are important class of structures, which occupy a major space in the domain of natural and bioactive compounds. For this reason, development of new synthetic strategies for their controllable synthesis became of special interests. The development of novel photoredox systems with wide-range application in organic synthesis is particularly interesting. Organic dyes have been widely applied as photoredox catalysts in organic synthesis. Their low costs compared to the typical photocatalysts based on transition metals make them an excellent alternative. This review describes proceedings since 2015 in the area of application of metal-free, visible-light-mediated catalysis for assembling various heterocyclic scaffolds containing five- and six-membered rings bearing nitrogen and oxygen heteroatoms.

## 1. Introduction

**Figure 1:**
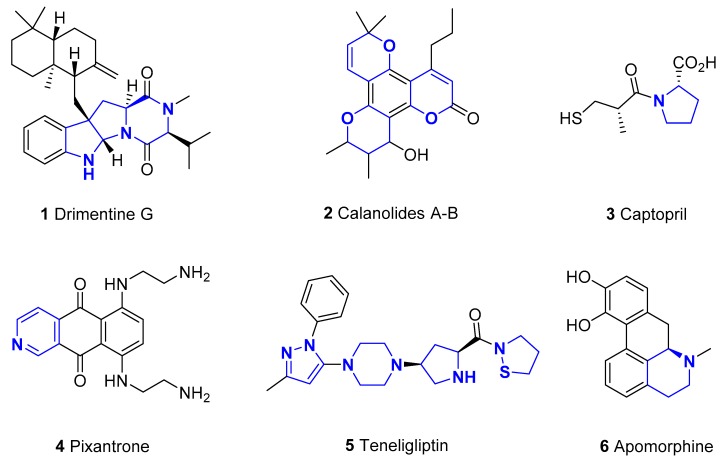
Selected example of current drugs containing heterocyclic scaffold.

Heterocycles are a very important class of structural motifs that can be found in many pharmaceuticals and natural products [1,2,3]. Numerous of them contain five- or six-membered rings bearing different heteroatom, like nitrogen or oxygen. Drimentine G, for example, is composed of three heterocyclic rings and indicates anticancer and antibacterial activities; others like Captopril is an ACE inhibitor used for the treatment of hypertension (Figure 1) [4,5,6,7]. As a consequence of potent bioactivity of many heterocycles, significant efforts have been devoted toward the development of new synthetic strategies for their preparation [3].

During the last years, application and development of visible-light-mediated catalysis in organic chemistry became a topic of immense importance [8,9]. It is due to the fact that opposite to many reactions, a light source is used to generate reactive species without the need to apply stoichiometric activators [10,11]. To fulfill the needs of the solar energy usage, structurally diverse compounds based on transition metals have been developed [12]. To pursue the ideal chemical transformation, light-emitting diodes (LEDs) or compact fluorescent lamps (CFLs) are used as a light source because of their low costs, reasonable energy consumption, and general availability.

**Figure 2:**
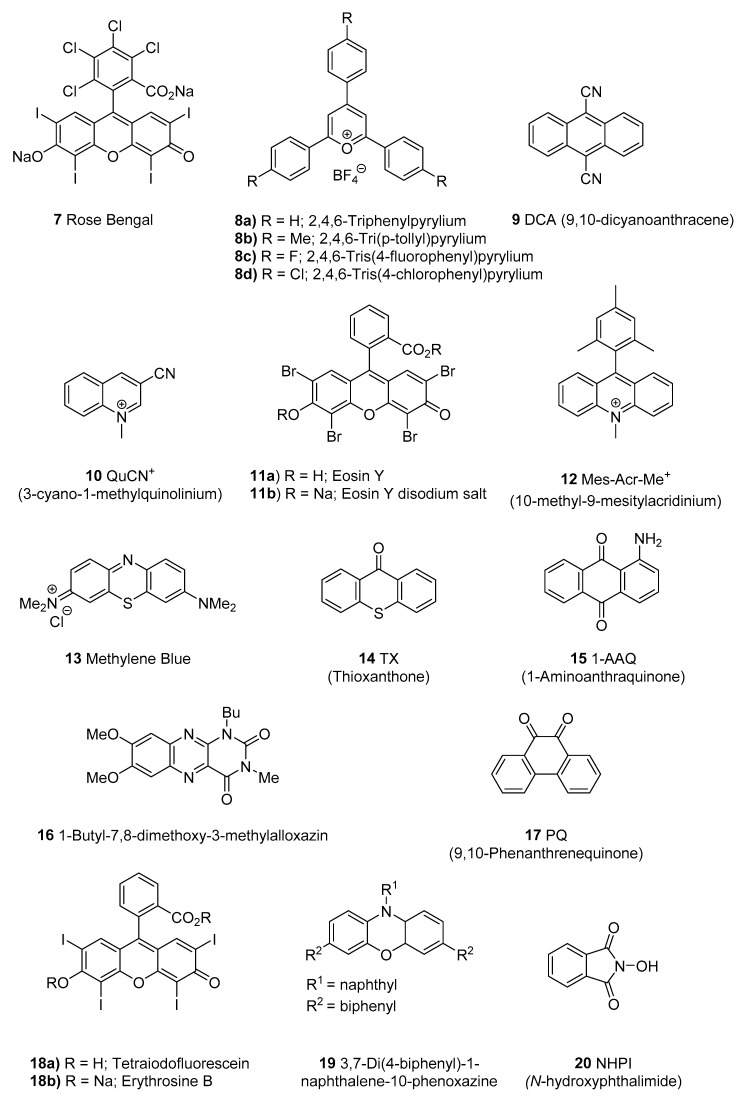
Examples of metal-free photoredox catalysts.

To create heterocyclic scaffolds, comprehensive photocatalytic strategies based on various metal complexes have been already disclosed [13,14,15,16,17,18,19,20]. However, their metal-free analogues play a significant role in terms of green chemistry [21] and medicine because of the high toxicity of many transition-metal complexes [22,23,24]. Organic dyes capable of visible-light-spectra absorption are generally less expensive and less toxic compared to the classical iridium or ruthenium catalysts. Thanks to high air stability, they are also easy to handle. For this reason, organic dyes act as an attractive alternative to transition-metal complexes [25,26,27]. Figure 2 shows the examples of most commonly used metal-free catalysts in photoredox chemistry.

This article presents the coverage of the recent advances in the application of metal-free, visible-light-mediated catalysis for assembling five- and six-member heterocyclic scaffolds containing nitrogen and oxygen heteroatoms. We are mainly focusing on the new metal-free photochemical reactions discovered after the year 2015 in the synthesis of aromatic and non-aromatic heterocycles. The article is organized into three chapters, which describe synthetic strategies for the preparation of nitrogen-containing heterocycles, oxygen-containing heterocycles, and heterocycles containing more than one heteroatom.

## 2. Application of Visible-Light-Mediated Catalysis in Synthesis of Heterocyclic Compounds

Many organic compounds isolated from nature contain five- or six-membered heterocycle scaffolds. These compounds are very interesting due to their potent bioactivities and applications as therapeutic drugs. For example, many heterocyclic derivatives, like emetine, papaverine, theophylline, etc. containing heterocyclic scaffold are used as life-saving pharmaceuticals [28]. Moreover, heterocyclic systems very often act as suitable intermediates in the synthesis of more complex molecules [29]. As part of their well-defined structure, they may contain *N*-, *O*-, *S*-, or other heteroatoms. Among the reaction strategies that lead to heterocycles, many of them use heavy metals, or require harsh reaction conditions [30]. Therefore, development of more sustainable approaches for constructing highly functionalized heterocycles have gained a considerable interest.

### 2.1. N-containing Heterocycles

#### 2.1.1. Aromatic Heterocycles

**Scheme 1:**
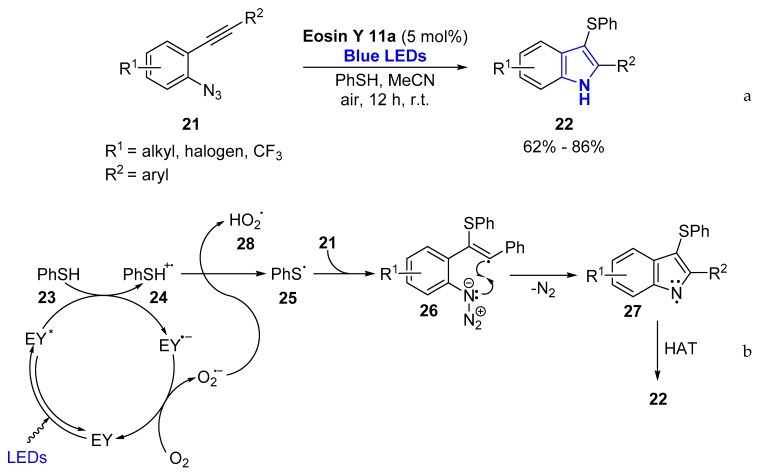
(**a**) Vicinal thioamination of alkynes, mediated by Eosin Y; (**b**) Proposed mechanism for vicinal thioamination of alkynes, mediated by Eosin Y.

Nitrogen bearing heterocycles constitute the majority in the field of heterocyclic chemistry. Various biologically active natural or synthetic products prevail the substituted indole motif. For example, some of the 2-substituted-indoles have been found as the successful leukotriene-modifier drugs in cardiovascular disease [31], others gain therapeutic interest in the treatment of cancer, HIV, heart disease, allergies, etc. [32]. To construct functionalized indoles, Kshirsagar and co-workers disclosed a photoredox-catalyzed vicinal thioamination of alkynes [33]. In this context, Eosin Y **11a** catalyzed radical cascade annulation generates various 3-sulfenylindoles **22** in up to 86% yield (Scheme 1a). This metal and strong-oxidant-free synthesis is based on the proposed mechanism presented in Scheme 1b. Eosin Y **11a** (EY) is first excited by the blue LED irradiation to EY*, which is then reduced to EY^•−^ by oxidizing thiophenol to the radical species **24**. Re-oxidation of EY^•−^ to its ground state takes place by air oxygen producing O_2_^•−^ species. Deprotonation of thiophenol cation radical **24** by the O_2_^•−^ gives hydroperoxyl radical **28** and radical **25**, which undergoes addition to the triple bond of **21** and produces another radical **26**. Subsequent intramolecular cyclization followed by N_2_ release delivers intermediate radical **27**. Finally, hydrogen atom transfer (HAT) from hydrogen source present in the reaction mixture gives product **22**.

**Scheme 2:**
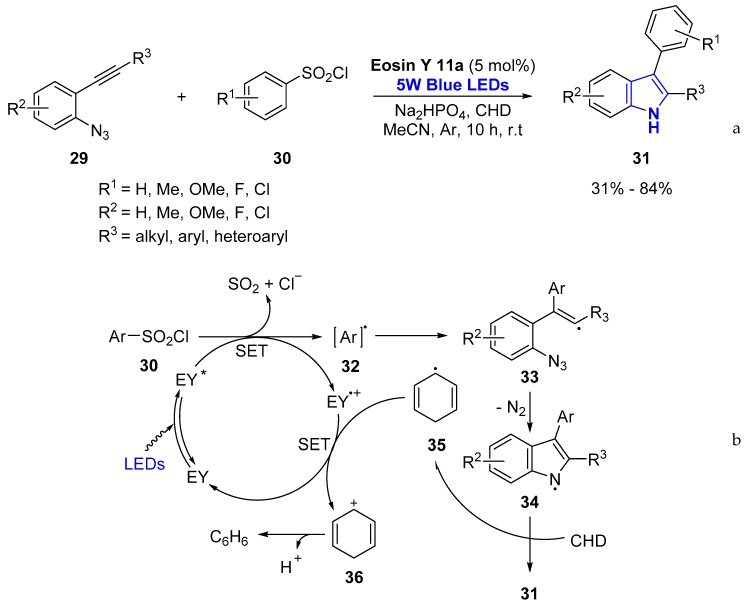
(**a**) Visible-light-induced cyclization of arylsulfonyl chlorides; (**b**) Proposed mechanism.

2,3-Disubstituted indoles can be also obtained by cyclization of arylsulfonyl chlorides with *ortho*-azidoarylalkenes [34]. This transition-metal free process described by Gu group, emerges as an efficient protocol, exhibiting high functional-group tolerance. Various indoles **31** can be prepared in up to 84% yield (Scheme 2a). Mechanism of this annulation is shown in Scheme 2b. Excited Eosin Y **11a** (EY) is responsible for reduction by SET of benzenesulfonyl chloride **30** to aryl radical **32** and EY^•+^. Formed phenyl radical **32** undergoes addition to the triple bond of **29** forming radical intermediate **33**, which subsequently undergoes intramolecular cyclization with the azide group to *N*-radical intermediate **34** with extrusion of nitrogen. Finally, H-atom abstraction from cyclohexadiene (CHD) by **34** forms desired product **31** and CHD radical **35**, which closes the photoredox cycle by SET process regenerating Eosin catalyst in its ground state (Scheme 2b).

Fluorine-containing compounds can often be found in medicinal chemistry due to improvement bioavailability, lipophilicity, or the metabolic stability compared to their non-fluorinated analogs [35]. For this reason, the synthesis of fluorinated heterocycles is particularly interesting.

**Scheme 3:**
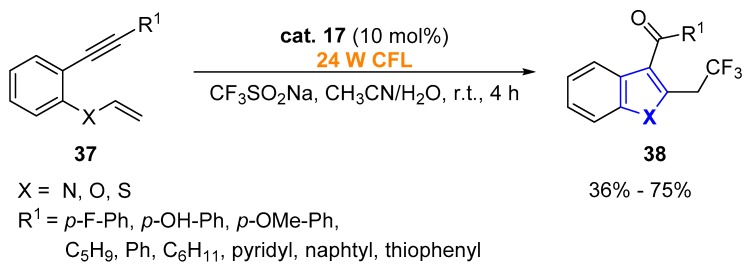
Dehydrogenative cascade trifluoromethylation and oxidation of 1,6-enynes.

The intramolecular cyclization of various 1,6-enynes **37** can be found as a general and powerful strategy in the synthesis of indole derivatives. In this context, Kumar and co-workers proposed metal and oxidant-free visible-light-induced trifluoromethylation of alkynes **37** (Scheme 3), using 9,10-phenanthrequinone catalyst **17** under compact fluorescent lamp (CFL) irradiation [36].

In addition, the described protocol is also effective for constructing benzofuran or thiophene frameworks. CFL irradiation of the CF_3_SO_2_Na derivative generates the initiating CF_3_ radical, which undergoes selective addition to the double bond acceptor, while subsequent intramolecular cyclization gives final aromatic derivatives **38** in up to 75% yield.

**Scheme 4:**
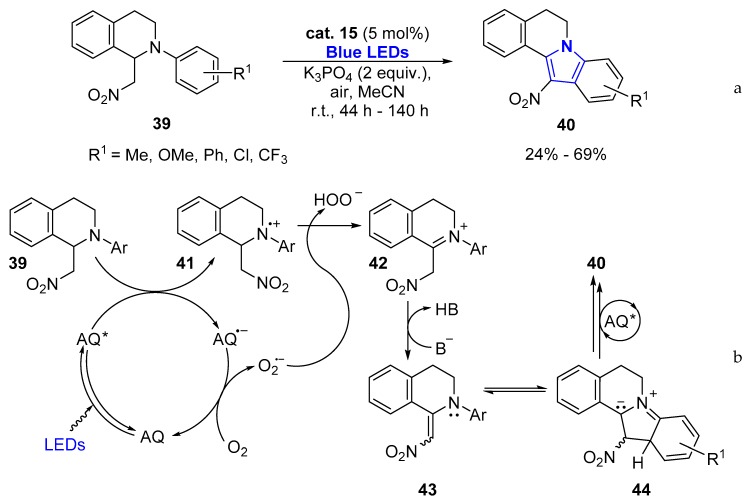
(**a**) A visible-light-induced cross-dehydrogenative cascade in the synthesis of tetrahydroisoquinoline derivatives; (**b**) Proposed mechanism.

The cascade-type reactions have been established as a general strategy for the synthesis of highly functionalized compounds [37]. In this context, Brasholz and co-workers described a visible-light-induced photocatalytic cascade reaction in the synthesis of indoloisoquinolines **40** using amino-substituted anthraquinone **15** as photocatalyst [38]. Formation of new heterocyclic ring is based on the dehydrogenation–cyclization–oxidation cascade that converts tetrahydroisoquinolines **39** into substituted tetracyclic heterocycles **40** (Scheme 4a). A plausible reaction mechanism starts on electron transfer between amine **39** and the photoinduced anthraquinone catalyst **15** (AQ*) with the formation of amine radical cation **41**. Hydrogen atom abstraction from **41** by superoxide radical anion converts amine **41** into iminium ion **42**. Subsequent deprotonation of **42** gives nitroenamine **43**, which undergoes electrocyclic ring closure to ylide **44**. Finally, rearomatization followed by photoinduced catalytic oxidation leads to appropriate 12-nitroindoloisoquinoline **40** in up to 69% yield (Scheme 4b).

**Scheme 5:**
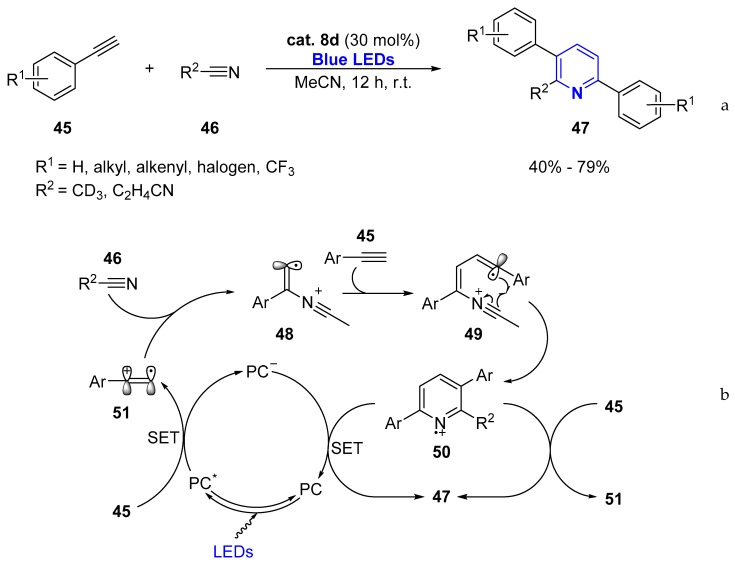
(**a**) [2 + 2 + 2] Cyclization of alkynes with nitriles; (**b**) Proposed mechanism.

Pyridine core is widespread in many bioactive compounds [1,2,3]. The versatile methodology employing visible-light-promoted [2 + 2 + 2] cyclization of alkynes with nitriles to pyridines recently has been described by Wang and Meng group [39]. Using pyrylium salt **8d** as photoredox catalysts, various 2,3,6-trisubstituted pyridines **47** can be prepared under blue LED irradiation. A wide range of functional group tolerance makes various pyridines **47** accessible in up to 79% yield (Scheme 5a). In Scheme 5b, a reasonable mechanism for this transformation is suggested. Blue LED irradiation of the pyrylium salt **8d** (PC) in the presence of phenyl acetylene **45** generates the radical cation **51**, which reacts with the nitrile **46** and generates intermediate **48**. Subsequent addition of **48** to another phenyl acetylene molecule **45** affords intermediate **49**, which undergoes intramolecular cyclization to pyridine radical cation **50**. Finally, reduction of **50** by pyrylium anion (PC^−^) via a SET process provides the product **47** and regenerated catalyst (PC) in its ground state.

**Scheme 6:**
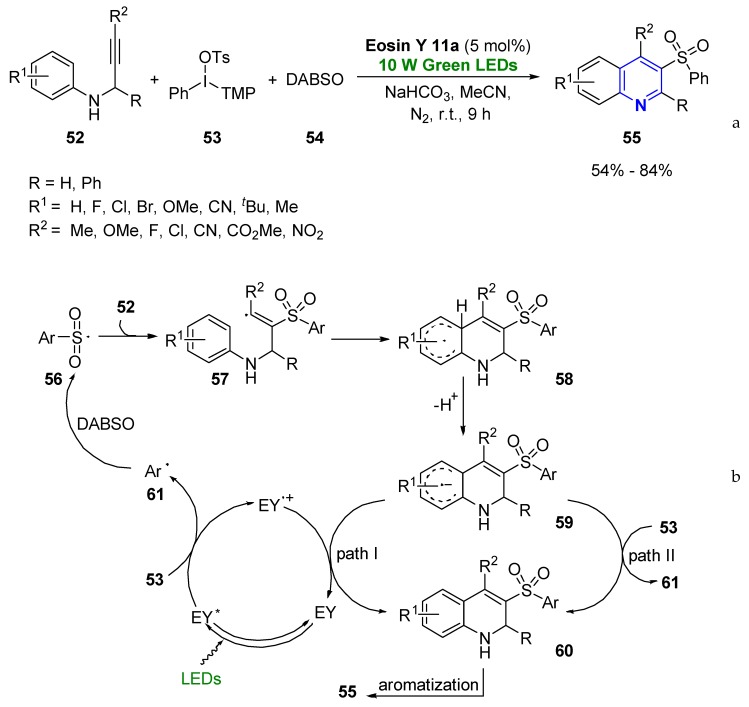
(**a**) Cycloaddition of *N*-propargyl aromatic amines, diaryliodonium salts, and sulfur dioxide; (**b**) Proposed mechanism.

Recently, visible-light-photoredox catalysis has been used for the synthesis of various quinolones as well. The Zhang group developed an impressive example of *N*-propargyl aromatic amine application in the synthesis of 3-arylsulfonylquinolines **55** (Scheme 6a). The authors demonstrated a visible-light-induced multicomponent cascade cycloaddition of **52** with diaryliodonium salts **53** and sulfur dioxide [40]. This three-component reaction can be achieved using Eosin Y **11a** catalysts under irradiation by green LEDs in the presence of DABSO (DABCO·(SO_2_)_2_) as sulfur dioxide source. This transformation performs effectively with a wide functional group tolerance in up to 84% yield.

On the basis of a series of control experiments, the authors proposed a plausible catalytic cycle (Scheme 6b). First, irradiation of Eosin Y **11a** by green LEDs generates excited Eosin Y (EY*), which undergoes oxidative quenching with iodonium salt **53** to appropriate aryl radical **61** and EY^•+^. Subsequent reaction of aryl radical **60** with DABSO generates sulfonyl radical **56**, which react regioselectively with the triple bond of **52** forming alkenyl radical **57**. Intramolecular cyclization of **57** produce annulated aryl radical **58**. Deprotonation of **58** generates another radical **59**, which is oxidized to **60** by EY^•+^ thus regenerating eosin catalyst in its ground state. Alternatively, radical **59** can be oxidized by **53** leading to the dihydroquinoline derivative **60** (path II). Finally, sulfonated 1,2-dihydroquinoline **60** undergoes dehydroaromatization to the product **55**.

**Scheme 7:**
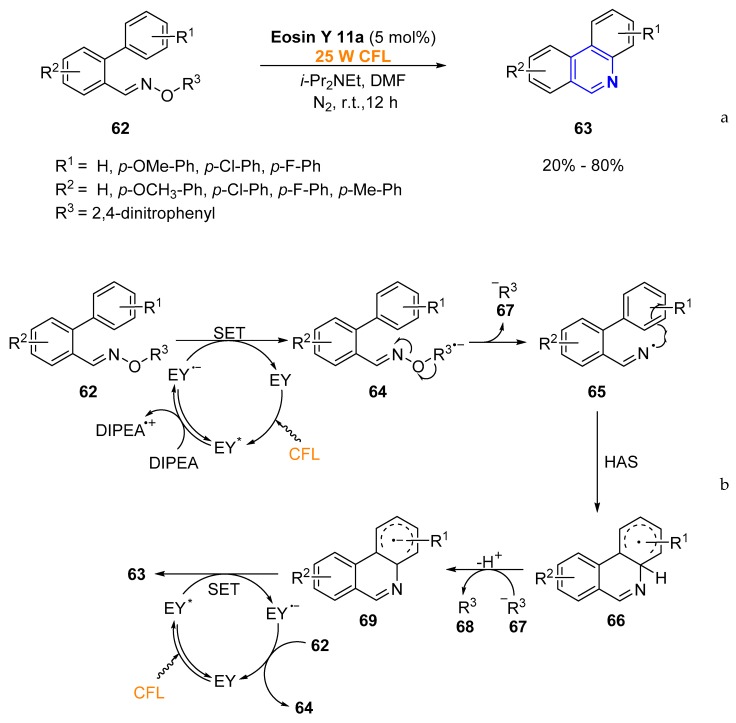
(**a**) Cyclization of O-(2,4-dinitrophenyl)oximes to phenanthridine; (**b**) Proposed mechanism.

As an efficient strategy for the construction of phenanthridine skeleton, the radical cyclization involving an iminyl radical is often invoked [41]. To develop more efficient and greener approach, Xie group reported visible-light-mediated cyclization of O-2,4-dinitrophenyl oximes **62** to phenanthridine **63** using Eosin Y **11a** under CFL irradiation (Scheme 7a) [42]. The authors proposed a plausible catalytic cycle for this valuable process (Scheme 7b). Firstly, excited Eosin EY* is reduced by (*i*-Pr)_2_NEt to Eosin EY^•−^, which undergoes single electron transfer (SET) with **62** and generate radical anion **64**.

Fragmentation of the intermediate **64** followed by intramolecular cyclization gives radical **66**, which is then deprotonated by phenoxyl anion **67** to radical anion **69**. Formed intermediate **69** undergoes another photocatalytic cycle with the excited state of eosin by SET leading to the final product **63** in moderate to good yields (up to 80%).

**Scheme 8:**
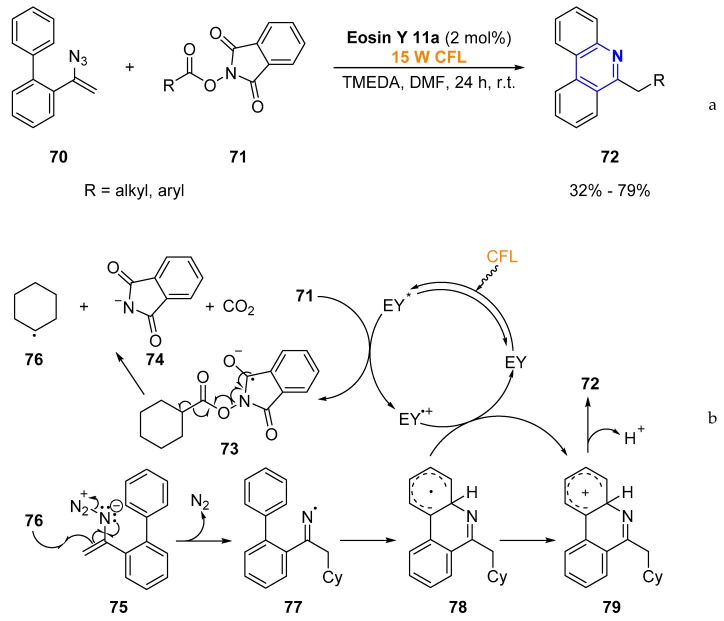
(**a**) Decarboxylative radical cyclization of vinyl azides with *N*-acyloxyphthalimides; (**b**) Proposed mechanism.

In a recent investigation, Guo applied decarboxylative cyclization of *N*-acyloxylphthalimides **71** with vinyl azides **70** in the synthesis of various phenanthridine **72** [43]. This tandem radical addition/cyclization process affords substituted products in up to 79% yield (Scheme 8a). The authors proposed a possible mechanism for this transformation (Scheme 8b). Firstly, irradiation of Eosin Y **11a** by CFL affords the excited Eosin Y catalyst (EY*). Subsequent single electron transfer with **71** generates radical anion **73**, which undergoes fragmentation to CO_2_, phtalimide anion **74,** and radical **76**. Addition of **76** to the double bond of **75** followed by extrusion of nitrogen produce iminyl radical **77**, which undergoes series of transformations: intramolecular cyclization, oxidation by Eosin Y^•+^, and deprotonation to the final product **72**.

#### 2.1.2. Non-Aromatic Heterocycles

Non-aromatic heterocycles are an integral part of heterocycles’ family. Same as their aromatic analogues they are widely distributed in nature, therefore significant efforts have been devoted to their synthesis [1,2,3].

**Scheme 9:**
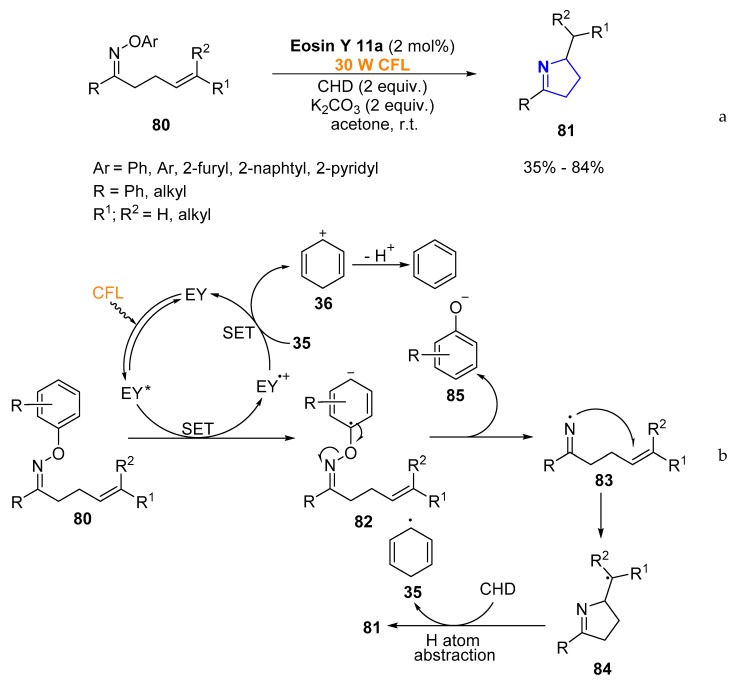
(**a**) Hydroimination and iminohydroxylation cyclization of olefins; (**b**) Proposed mechanism.

In a recent investigation, Leonori reported a novel visible-light-mediated generation of 5-membered cyclic imines **81** [44]. Reaction is based on the hydroimination and iminohydroxylation cyclization of olefins **80** using Eosin Y **11a** as a photoredox catalyst (Scheme 9a). In this process, non-aromatic five heterocyclic ring is formed in up to 84% yield. Based on experimental findings, a plausible reaction mechanism is depicted in Scheme 9b. The suggested mechanism is based on single electron transfer from the enhanced Eosin catalyst (EY*) to the starting material **80** with production of anion radical **82**. Radical **82** undergoes fragmentation to phenoxide **85** and radical intermediate **83**. Next, radical **83** undergoes 5-*exo*-trig cyclization to the five-membered *N*-heterocycle radical **84**. Finally, hydrogen abstraction from **84** by cyclohexadiene (CHD) leads to the desired product **81** and CHD radical **35**, which closes the photoredox cycle by SET process regenerating Eosin catalyst in its ground state.

**Scheme 10:**
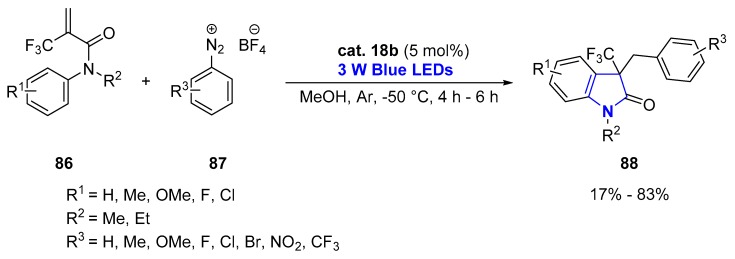
Synthesis of 3-(trifluoromethyl)indolin-2-one derivatives under photoredox conditions.

Cyclization of *N*-arylacrylamides **86** have been found as a useful strategy in the synthesis of indolin-2-one compounds **88**. Novák and Tóth group developed cyclization of trifluoromethylated acrylamides under visible-light conditions catalyzed by Erythrosine B **18b** (Scheme 10) [45]. The elaborated arylation/cyclization sequence is initiated by aryl radical generation from diazonium salt **87**, which undergoes addition to the double bond of **86** followed by radical cyclization. In this process, structurally different heterocyclic units **88** can be prepared in up to 83% yield.

**Scheme 11:**
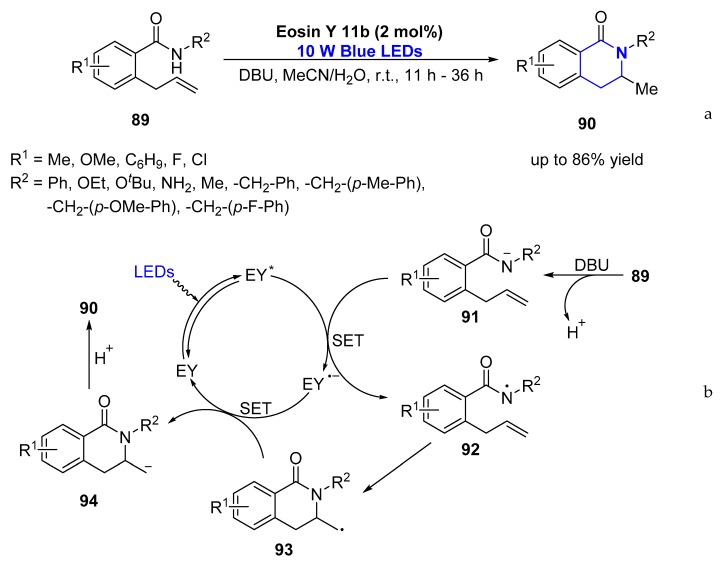
(**a**) Synthesis of 3,4-dihydroisoquinolinones; (**b**) Proposed mechanism.

Various dihydroisoquinolin derivatives can be obtained by using Eosin Y **11b** catalyst under visible-light conditions [46]. Huang group showed that substituted 3,4-dihydroisoquinolinones **90** can be received in a very good yields by intramolecular cyclization of appropriate amides **89** (Scheme 11a). Mechanistic pathway for this transformation is presented in Scheme 11b. It starts by the formation of excited state of Eosin catalyst (EY*) and deprotonation of **89** by DBU with anion **91** formation. Single electron transfer between anion **91** and EY* leads to the radical **92**. Subsequent intramolecular cyclization followed by another SET cycle recovers ground state of Eosin catalyst together with the anion **94** formation, which undergoes protonation to the final product **90**.

**Scheme 12:**
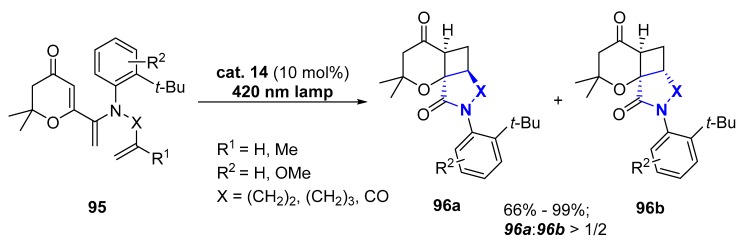
[2 + 2] Photocycloaddition of enones.

Throughout the past decades, stereoselective synthesis became of special interest, both by academia and industry scientists [47]. Recently, Sivaguru group [48] demonstrated that heterocycles possessing multiple stereocenters can be prepared using photochemical chemistry by intramolecular [2 + 2] photocycloaddition (Scheme 12). By incorporating axial chirality into the system, cycloaddition process can be effectively controlled leading to the appropriate stereoisomer **96** in up to 99% yield.

**Scheme 13:**
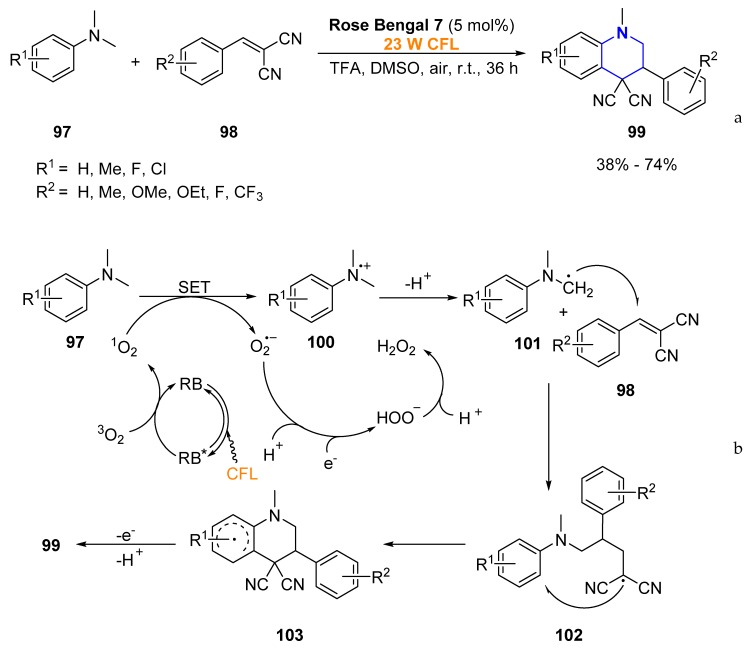
(**a**) Synthesis of tetrahydroquinoline derivatives; (**b**) Proposed mechanism for synthesis of tetrahydroquinoline derivatives.

Tetrahydroquinolines (THQ) are an important class of compounds being significant synthetic target for chemists. In this context, Guan and co-workers reported synthesis of tetrahydroquinoline derivatives **99** utilizing Rose Bengal **7** (RB) as a catalyst under irradiation by the compact fluorescent lamp (CFL) [49]. This newer approach to the previously reported by Rueping and coworkes [50] proceeds via tandem radical cyclization of *N*,*N*-dimethylanilines **97** with 2-benzylidenemalononitriles **98** to six-membered non-aromatic heterocycles **99** in up to 74% yield (Scheme 13a). The mechanism of this transformation can be rationalized on the basis of ^1^O_2_ formation by interaction of O_2_ with the excited-state of Rose Bengal catalyst (RB*) (Scheme 13b). Generated singlet oxygen proceeds single electron transfer with *N*,*N*-dimethylaniline **97** with the formation of cation radical **100**. Absorbing proton by dioxygen radical anion results in the formation of amine radical **101**, which react with 2-benzylidenepropanedinitrile **98** and produce the alkyl radical **102**. Finally, intramolecular cyclization of **102** followed by rearomatization by the second electron transfer/proton elimination step gives the final product **99**.

**Scheme 14:**
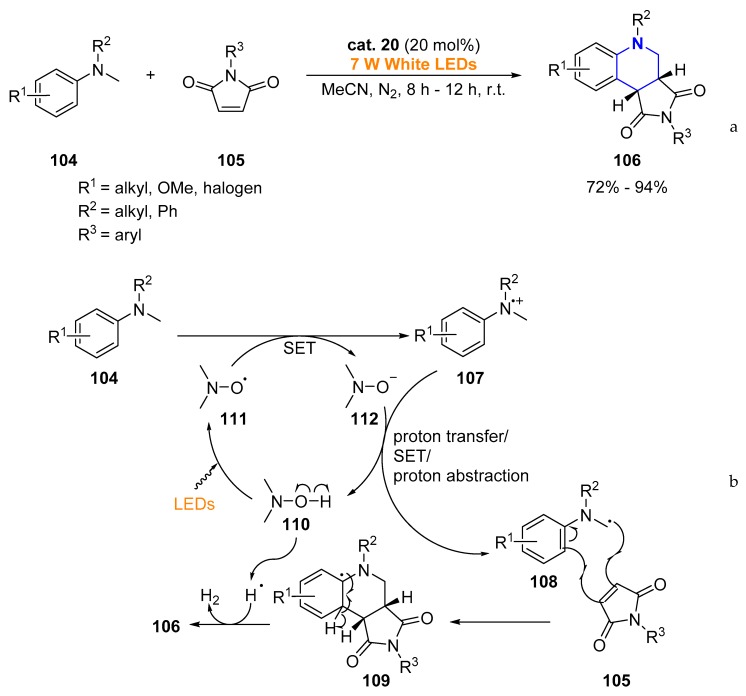
(**a**) [4 + 2] Radical cyclization of N-methylanilines with maleimides in the synthesis of tetrahydroquinolines; (**b**) Proposed mechanism.

Similarly, using (*N*,*N*-dimethyl)anilines **104** for the formation of α–amino radicals, Zhang [51] and Yadav [52] group revealed an efficient [4 + 2] cyclization to the THQ analogues **106**. In this process, *N*-hydroxyphthalimide **20** was used as an organophotoredox catalyst under white LEDs irradiation (Scheme 14a). Elaborated protocol involves C(sp^3^)-H activation of *N*-methylanilines **104** for the formation of α–amino radical **108**, which undergoes [4 + 2] cyclization with maleimide **105** to form intermediate **109**. Finally, aromatization of **109** leads to the desired product **106** in up to 94% yield (Scheme 14b).

**Scheme 15:**
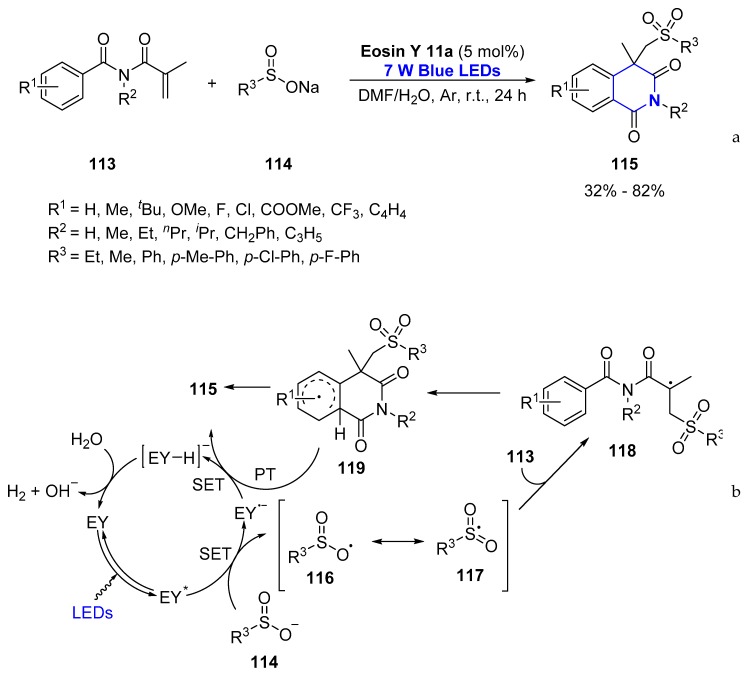
(**a**) Preparation of isoquinolinediones; (**b**) Proposed mechanism for preparation of isoquinolinediones.

By utilizing sulfonyl radicals, many sulfonyl containing compounds can be prepared using photoredox catalysis. The use of cheap, non-irritating sodium sulfinate opens the way to the possibility to generate sulfonyl radicals by means of visible-light-induced catalysis. Zuo et al. disclosed a tandem radical addition/cyclization concept for the synthesis of isoquinolinediones [53]. A mild radical cascade reaction is based on sulfonyl radical generation from the appropriate sodium sulfinate **114** and Eosin Y **11a** under blue LED irradiation (Scheme 15a). In this tandem approach, a broad range of substrates and functional group are tolerated leading to the desired products in up to 82% yield. The reaction starts on generation of excited state of Eosin (EY*) by blue LEDs irradiation which is reductively quenched by sulfinate **114** to sulfinate radical **116** and EY^•−^ catalyst (Scheme 15b). More stable form of radical **116**—radical **117** undergoes subsequent addition to the double bond of **113** generating radical **118**, which yields radical **119** by intramolecular cyclization. Finally, the single electron and proton transfer between EY^•−^ and **119** delivers the desired product **115**. The photocatalytic cycle is completed by the reaction of Eosin hydride with proton, which leads to the release of hydrogen from the reaction mixture.

### 2.2. O-Containing Heterocycles

#### 2.2.1. Aromatic Heterocycles

**Scheme 16:**
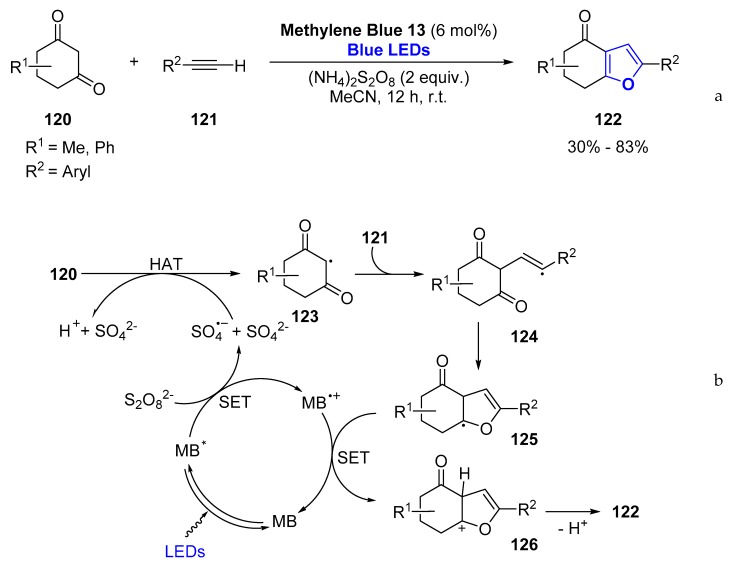
(**a**) Visible-light-mediated oxidative [3 + 2] cycloaddition in the synthesis of furans; (**b**) Proposed mechanism.

Similar to *N*-containing heterocycles, their oxygen analogues can be also found in many natural products and pharmaceuticals [54,55], attracting broad interests by scientific community. A new route toward the synthesis of a functionalized furan ring has been recently disclosed by Lei and co-workers [56]. Lei elaborated the synthesis of this useful scaffold through the visible-light-mediated oxidative [3 + 2] cycloaddition of enols and alkynes **121** (Scheme 16a). This highly selective method of constructing substituted furans is based on the usage of Methylene Blue **13** as catalyst in combination with (NH_4_)_2_S_2_O_8_ as a terminal oxidant. General mechanism for this transformation is disclosed in Scheme 16b. First, visible-light irradiation of Methylene Blue (MB) leads to the formation of its excited-state MB*. The persulfate is then reduced by MB* to form the sulfate radical anion. Subsequent hydrogen atom transfer between **120** and sulfate radical anion forms intermediate **123**, which undergoes intermolecular addition to the triple bond of alkyne **121** with the formation of radical **124**. Next, intramolecular radical addition to the carbonyl oxygen forms a five-membered O-heterocycle **125** radical. Finally, oxidation of **125** by MB^•+^ followed by deprotonation leads to the final product **122**.

#### 2.2.2. Non-Aromatic Heterocycles

**Scheme 17:**
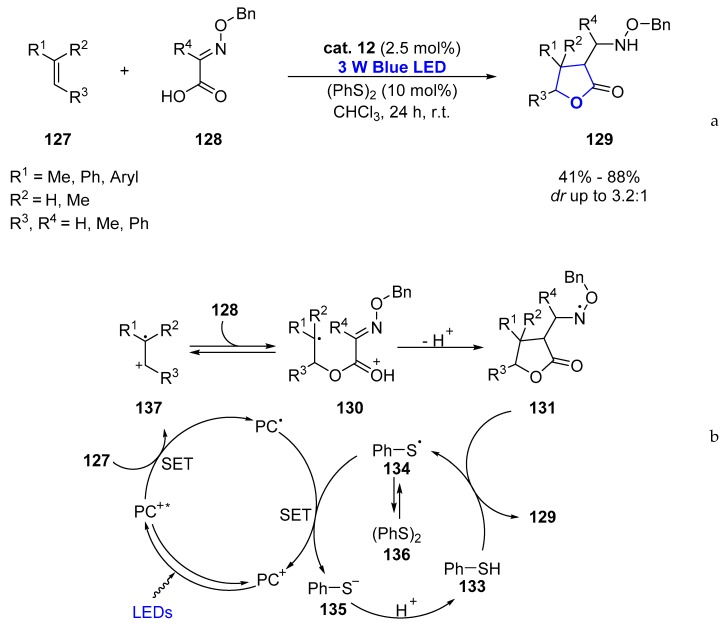
(**a**) Synthesis of 3,4-di-substituted mercaptolactones; (**b**) Proposed mechanism.

In addition to heteroaromatic compounds, their non-aromatic oxygen-containing analogues are equally important. Butyrolactones are a class of heterocycles highly prevalent in nature, indicating many interesting bioactivities [57,58]. Given their importance, many synthetic strategies for their preparations have been elaborated, including photoredox catalysis. In 2015, Nicewicz group presented a synthesis of α-benzyloamino-γ-butyrolactones **129** via polar radical crossover cycloaddition reaction [59]. The photoredox reaction is carried out using the Fukuzimi acridinium **12** photooxidant, easily accessible oxime acids **128** and alkenes **127**. Using given methodology, various lactones **129** with high functional group variations are accessible in very good yields (Scheme 17a). On the basis of the author’s previous work, they proposed a plausible reaction pathway for this interesting transformation (Scheme 17b) [60]. First, a single electron oxidation of the alkene **127** by the excited state of catalyst (PC^+^*) affords cation radical **137**. Next, the radical **137** reacts with the oxime acid **128** resulting in the formation of the radical **130**. Subsequent deprotonation of compound **130**, followed by intramolecular 5-*exo*-trig radical cyclization furnishes *N*-centered radical **131**. Finally, hydrogen atom transfer between **131** and **133** affords final product **129** and regenerates the acridinium catalyst **12** in its ground state. Authors indicated, however, that another mechanistic pathway without the use of H-atom donor co-catalyst might be involved.

The synthesis of structurally similar butyrolactones by metal-free visible-light-mediated catalysis has been also described by Liu [61] and Shah groups [62]. Moreover, by using amide analogues of **128** various γ-lactams or pyrrolidines can be prepared as well [63].

**Scheme 18:**
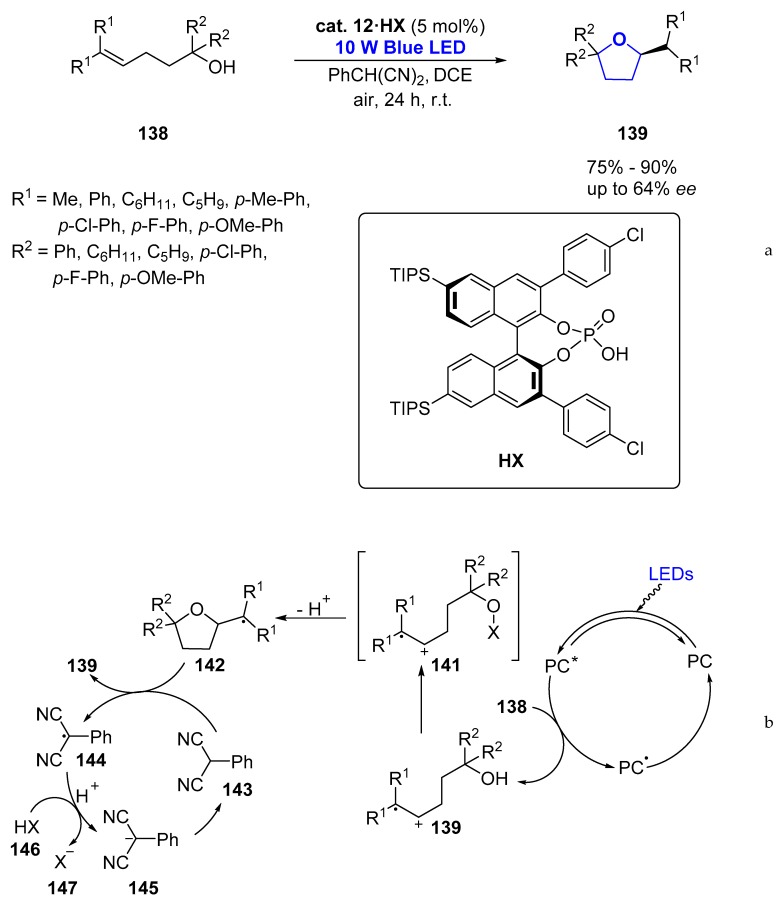
(**a**) Hydroetheryfication of alkenols; (**b**) Proposed mechanism.

An impressive example of a chiral ion-pair photoredox organocatalyst [64] in enantioselective hydroetheryfication of alkenols in stereoselective synthesis of tetrahydrofuran analogs has been recently disclosed by Luo and co-workers [65]. The described work showed that ion pair catalysts indicate improved activity compared to Fukuzimi catalysts **12** and proves that visible-light-mediated catalysis is feasible for stereoselective transformations (Scheme 18a). This reaction is based on the three stage process: single electron transfer, cyclization, and hydrogen transfer and has been described already in detail by Nicewicz group [60]. However, it has been disclosed by the authors that the origin of the higher activity observed with an ion pair catalyst is related with cyclization/H-transfer sequence. Phosphate anion in ion pair catalyst endows a longer lifetime of the chiral photocatalysts triplet state, introduces chirality, and assists in the H-shift (Scheme 18b).

**Scheme 19:**
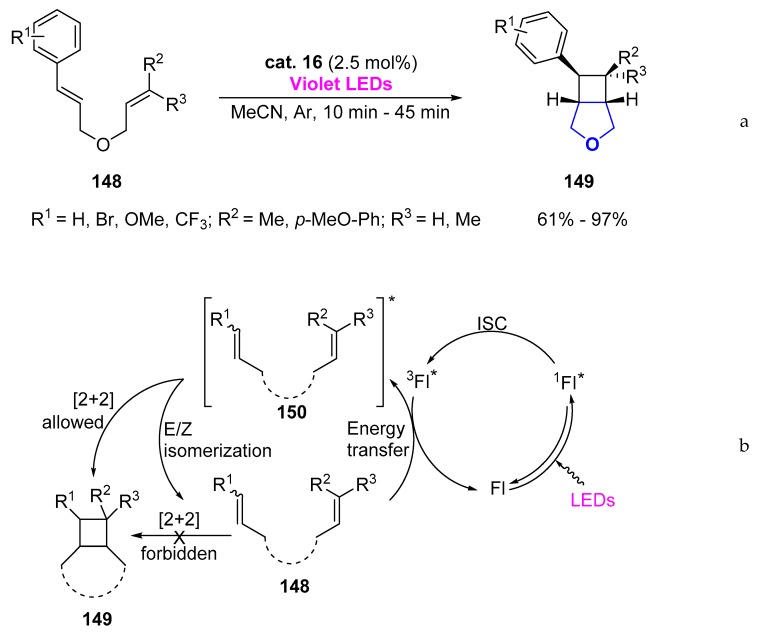
(**a**) Flavin-mediated visible-light [2 + 2] cycloaddition of dienes; (**b**) Proposed mechanism.

Cibulka group revealed that Flavin derivatives **16** can be a successful organic catalyst, able to perform intramolecular [2 + 2] cycloaddition of **148** under 400 nm violet LEDs irradiation [66,67,68] (Scheme 19a). 1-Butyl-7,8-dimethoxy-3-methylalloxazine **148** under irradiation undergoes intramolecular [2 + 2] cycloaddition of both styrene dienes forming bicyclic unit **149** (Scheme 19b). This photo-induced cycloaddition takes place with a broad spectrum of dienes leading to the formation of desired products **149** in good to excellent yields (up to 97%).

**Scheme 20:**
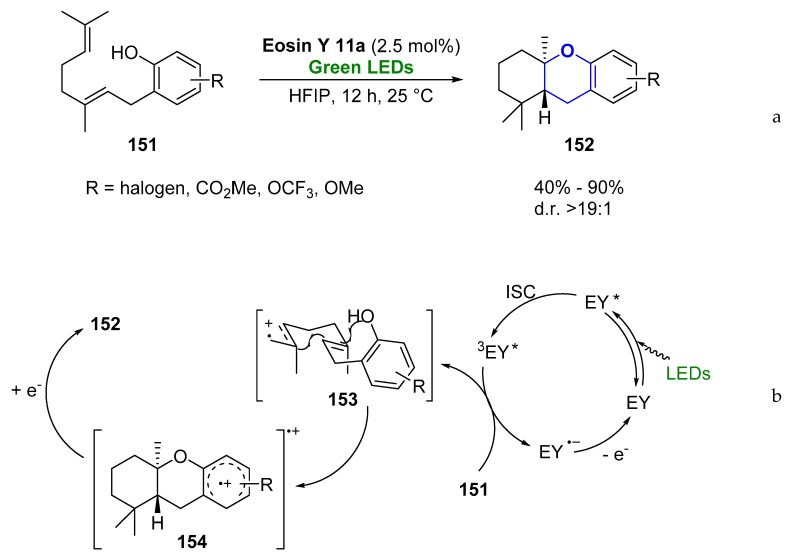
(**a**) Visible-light-mediated radical cascade cyclization of polyenes; (**b**) Proposed mechanism.

Ionic approach for polyene cyclization has been extensively explored, thus constituting the main synthetic strategy of polyene ring synthesis [69]. However, stereoselective radical cyclization can also have significant benefits [70]. In this context, Zhang and Luo group [71] demonstrated visible-light-induced cyclization of polyenes in the synthesis of various oxygen-containing heterocycles (Scheme 20a). The reported protocol is based on radical cascade cyclization using Eosin Y **11a** as a catalyst and green LEDs. Desired products **152** are prepared in moderate to excellent yields (up to 90%) and high stereoselectivities (d.r. > 19:1). In Scheme 20b, a reasonable mechanism for this transformation is suggested. First, excited state of Eosin catalyst (EY*) is formed upon green LEDs’ irradiation. Next, intersystem crossing (ISC) forms triplet ^3^EY*, which allows for electron transfer from the substrate **151** to the ^3^EY* resulting in the formation of the radical cation **153** and EY radical anion (EY^•−^). The generated radical **153** undergoes intramolecular radical cascade cyclization together with the hydrogen shift to the intermediate **154**. Finally, intermediate **154** is reduced to the desired product **152**, whereas Eosin radical anion (EY^•−^) is oxidized, regenerating catalyst and completing the photocatalytic cycle.

In addition to the sulfonated quinolines (Scheme 6), its oxygen containing analogs—cumarines can be also obtained. In this context, various alkynes undergoe arylsulfonylation with arylsulfinic acid and TBHP using Eosin Y **11a** as a catalyst. It is noteworthy that appropriate cumarines can be obtained in good yields with a wide functional group tolerance [72].

### 2.3. Heterocycles Bearing More Than One Heteroatom

**Scheme 21:**
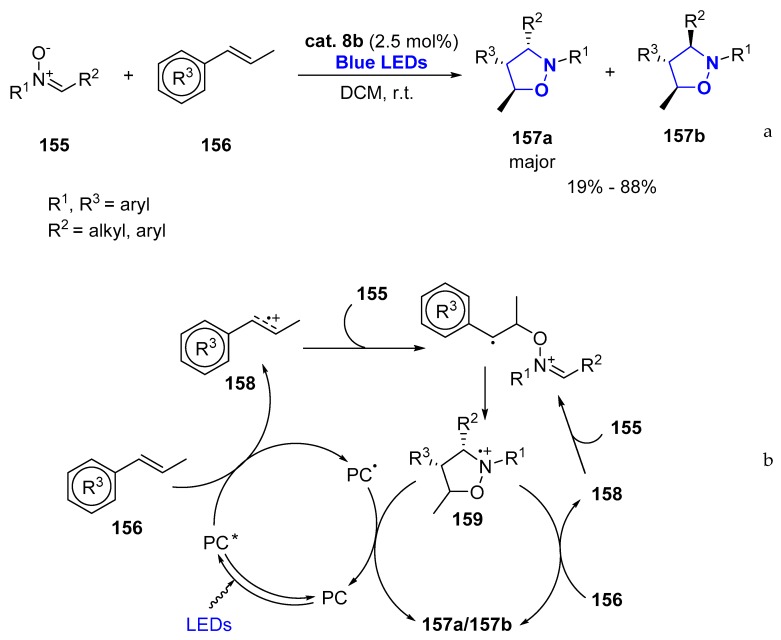
(**a**) Visible-light-mediated nitrone 1,3-dipolar cycloaddition; (**b**) Proposed mechanism.

An efficient and straightforward strategy for building functionalized, *N*- or *O*-bearing heteroatoms is the 1,3-dipolar cycloaddition [73]. Xia, Yang and collaborators [74] described visible-light-mediated *anti*-regioselective 1,3-dipolar cycloaddition of nitrones **155** with alkenes **156** (Scheme 21a). The nitrones are cyclized with styrenes and aliphatic alkenes via a polar radical crossover cycloaddition reaction using triphenylpyrrylium catalyst **8b** under blue light irradiation. This transformation is based on the mechanism presented in Scheme 21b. Initially, upon blue LEDs irradiation excited state of catalysts is formed (PC*), which then oxidize double bond of **156** via the reductive quenching process. Electrophilic addition of intermediate **158** to nitrone **155**, followed by a radical cyclization leads to the intermediate **159**. This intermediate act as an oxidant enabling regeneration of the catalyst by electron transfer alongside with the product formation. The authors also suggest a possible radical chain propagation between **158** and alkene **155**.

**Scheme 22:**
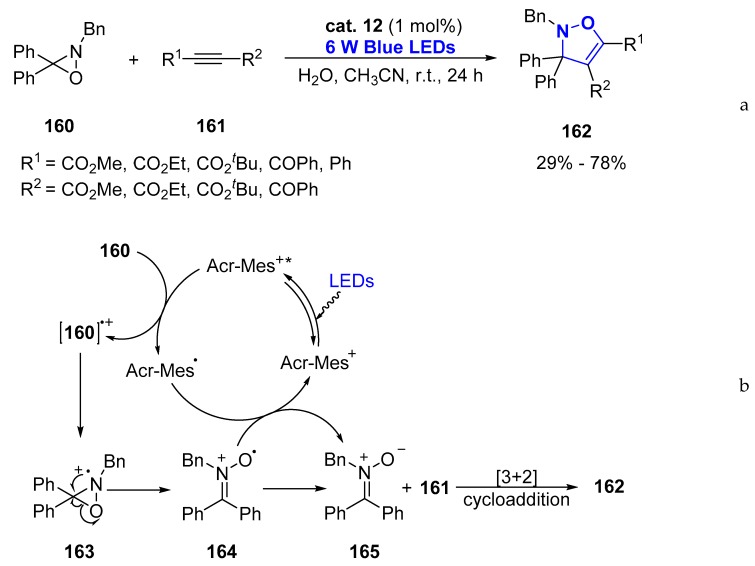
(**a**) [3 + 2] Cycloaddition of oxaziridines with alkynes; (**b**) Proposed mechanism.

Woo group [75] unveiled that nitrones can be also generated in situ from oxaziridines in photoredox conditions. They described synthetic method for the preparation of 4-isoxazolines **162** in a visible-light photoredox-catalyzed [3 + 2] cycloaddition of oxaziridines **160** with alkynes **161** (Scheme 22a). Described methodology relies on the in situ generation of nitrones from oxaziridines **160** by the single electron transfer process, which undergoes [3 + 2] cycloaddition with various alkynes. On the basis of a series of experiments, the following mechanism is proposed by the authors (Scheme 22b). Excited (Acr^+^-Mes*) catalyst formed upon blue LEDs irradiation allows for the single electron oxidation process of aziridine **160** alongside with the reduced photocatalyst (Acr^•^-Mes) formation. The radical cation **163** is then converted into the nitrone radical **164** through the ring opening process. Single-electron reduction of nitrone radical **164** by Acr^•^-Mes regenerates catalyst and forms nitrone **165**. Finally, [3 + 2] cycloaddition of nitrone **165** with alkyne **161** provides the product **162**.

**Scheme 23:**
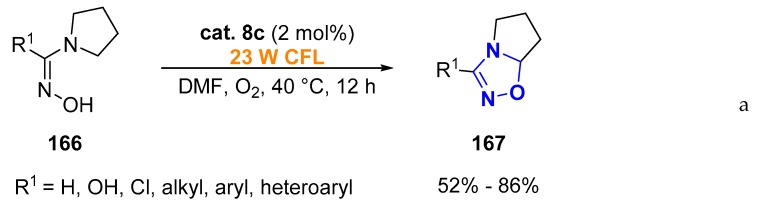

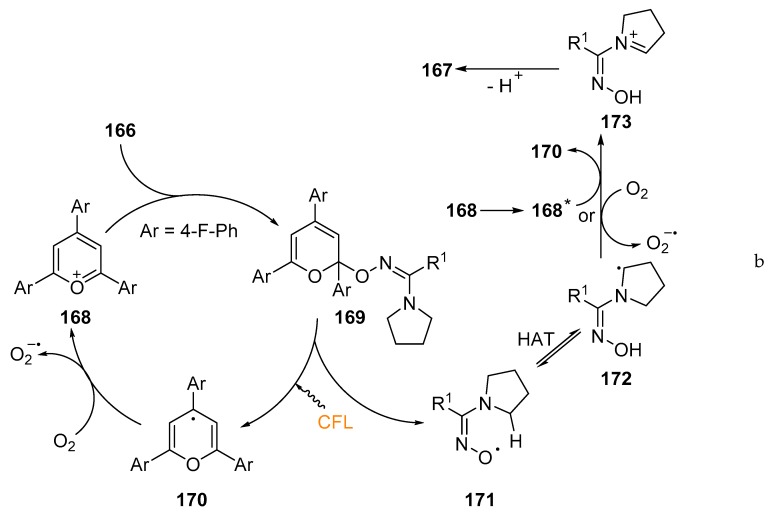
(**a**) Application of amidoximes in visible-light-driven synthesis of oxadiazolines; (**b**) Proposed mechanism.

1,2,4-Oxadiazole derivatives besides exhibiting biological activities have also found application in light-emitting diodes (OLEDs) [76]. Due to the importance of this group, Cho and co-workers developed oxidative cyclization of amidoximes under visible-light conditions [77]. Described protocol involves the intramolecular oxidative cyclization of amidoximes **166** in the presence of the triphenylpyrylium **8c** catalyst and molecular oxygen as the oxidant, promoted by compact fluorescent lamp (Scheme 23a). Opposite to all previous examples, in this particular transformation T(*p*-F)PPT act as both an electrophilic catalyst and a photocatalyst due to the fact that reaction works also in the dark, although less efficient. The authors proposed a possible mechanism for this transformation (Scheme 23b). Reaction starts by the nucleophilic addition of **166** to the triphenylpyrrylium ion **168** and generates intermediate **169**, which undergoes homolytic dissociation and produce two radicals: **171** and **170**. Additionally, this process is accelerated by the visible-light irradiation. The catalyst **168** is then regenerated by the molecular oxygen oxidation of **170** intermediate. Beside catalyst regeneration cycle, the radical **171** undergoes an intramolecular 1,5-hydrogen atom transfer (HAT) and produces the radical **172**. Subsequent oxidation of the compound **172** to the iminium ion by molecular oxygen or excited catalyst followed by intramolecular cyclization yields the final product **167**.

**Scheme 24:**
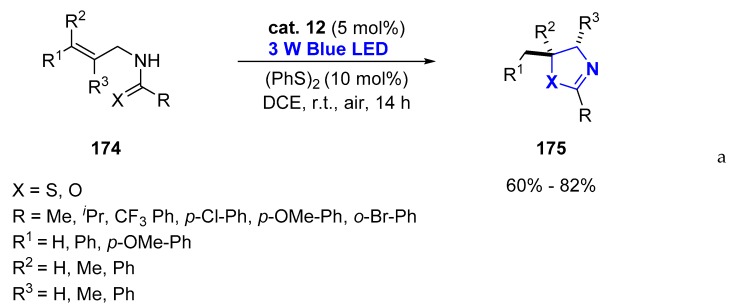

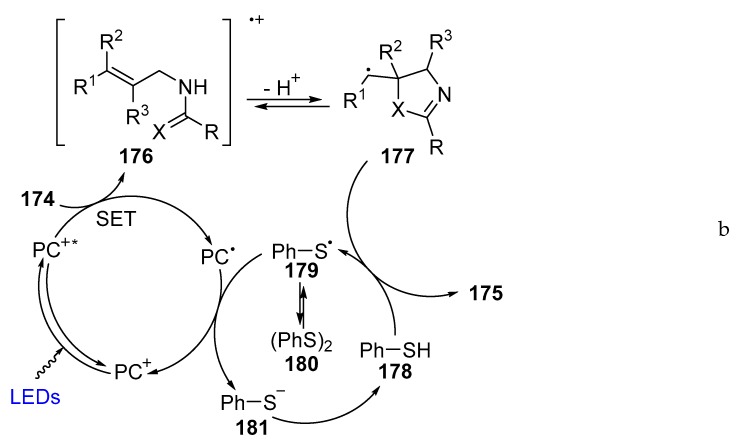
(**a**) Hydrofunctionalization of unsaturated amides; (**b**) Proposed mechanism.

Many natural or synthetic compounds containing oxazoline scaffold have been found to possess some level of interesting bioactivities [78]. Moreover, these structures are important building blocks in the syntheses of many chiral ligands in stereoselective synthesis [79]. Therefore, novel methodologies for the synthesis of these structural motifs are highly desirable. In this context, Nicewicz and co-workers disclosed photoredox-catalyzed hydrofunctionalization of unsaturated amides and thioamides in the synthesis of 2-oxazolines and 2-thiazolines [80]. This intramolecular functionalization is based on dual catalytic system comprised from **174** and phenyl disulphide (Scheme 24a).

This mild and efficient protocol leading to the products **175** in good yields (up to 82%) tolerates also many functional groups. In Scheme 24b, a reasonable mechanism for this transformation is suggested. Excitation of acridinium catalyst **12** by blue LED irradiation generates the active oxidant (PC^+^*), which accepts an electron from substrate **174**, generating cation radical intermediate **176**. Subsequent cyclization and proton loss affords cyclic radical intermediate **177**. Finally, hydrogen atom transfer from thiophenol **178** generates product **175** and a thiyl radical **179**. The thiyl radical **179** is presumed to re-oxidize the acridine radical catalyst (PC^•^), regenerating catalyst in its ground state.

**Scheme 25:**
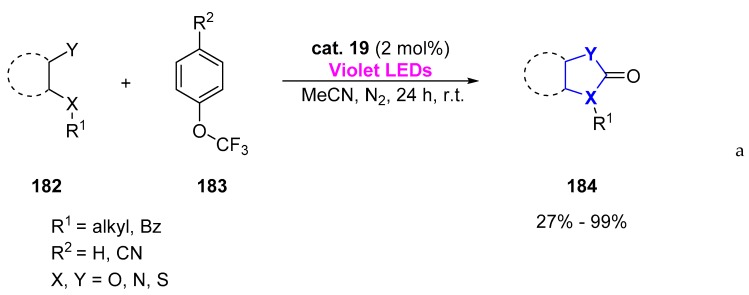

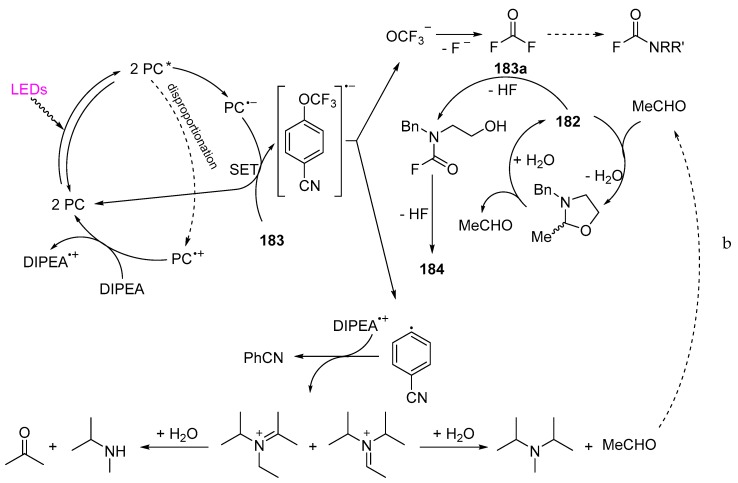
(**a**) Visible-light-mediated in situ generation and conversion of fluorophosgene; (**b**) Proposed mechanism.

An impressive example of visible-light-mediated generation and utilization of fluorophosgene was developed by König group [81]. Presented procedure is based on the cleavage of an aryl trifluoromethoxy ether **183** and in situ conversion of formed fluorophosgene for the synthesis of carbamates, carbonates, and urea derivatives **184** (Scheme 25a). The reported protocol is based on the single-electron reduction of 4-(trifluoromethoxy)benzonitrile **183** and its fragmentation into fluorophosgene **183a**, which undergoes further intramolecular cyclization with 1,2-diamines, aminoalcohols or diols **182** (Scheme 25b). In this way, carbonates, various carbamates, and urea derivatives **184** can be prepared in moderate to excellent yields.

**Scheme 26:**
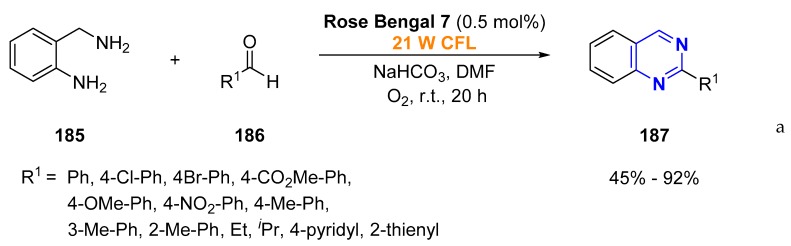

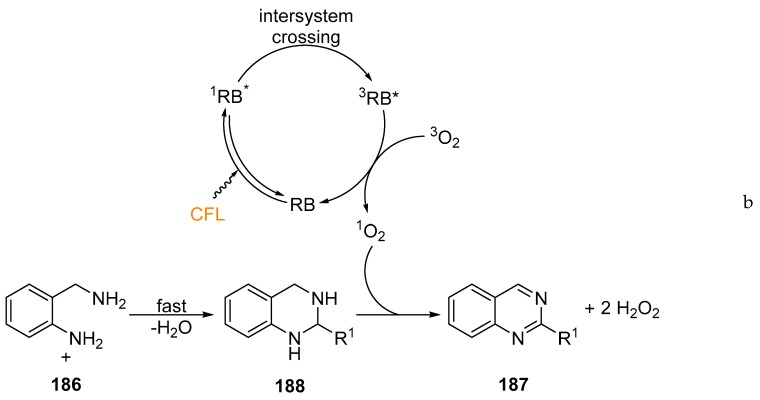
(**a**) Synthesis of quinazolines; (**b**) Proposed mechanism.

Quinazolines are another class of heterocyclic compounds extensively studied. Itoh et al. described the synthesis of quinazolines **187** under visible-light conditions by employing coupling of primary amines **185** and aldehydes **186** (Scheme 26a) [82]. A possible mechanism as proposed by the authors is disclosed in Scheme 26b. Initially, cyclization between the diamine **185** and the aldehyde **186** takes place leading to the diamine **188**. In the same time, Rose Bengal **7** (RB) is excited under irradiation followed by intersystem crossing (ISC) providing ^3^RB*. Subsequent energy transfer from triplet RB catalyst to oxygen produces reactive singlet oxygen ^1^O_2_ responsible for the oxidation of the compound **188** into the final product **187**.

**Scheme 27:**
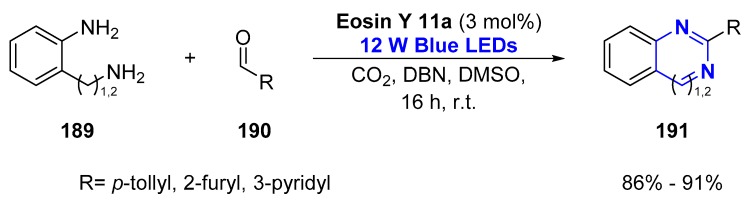
Dehydrogenation of amines to imines.

More recently, Das group also demonstrated cyclization of the diamines **189** and aldehydes **190** into heterocycles **191**, but in different reaction conditions [83] (Scheme 27). Opposite to the previous example, this protocol is based on CO_2_-catalyzed dehydrogenation/aromatization of amines under photoredox conditions. In this context, a corresponding amine formed upon cyclization of diamine **189** and aldehyde **190** is dehydrogenated and aromatized to the product **191**. A broad range of functional group can be used and desired products can be prepared in up to 91% yield. In addition to the diamine and aldehydes coupling, multisubstituted quinazolines and benzimidazoles can also be obtained by the radical cyclization of *N*-alkyl-*N*’-arylamidines catalyzed by Rose Bengal **7** in the presence of CBr_4_ and K_2_CO_3_. Tang group demonstrated that the given methodology tolerates a wide range of functional groups with a higher reaction efficiency [84].

**Scheme 28:**
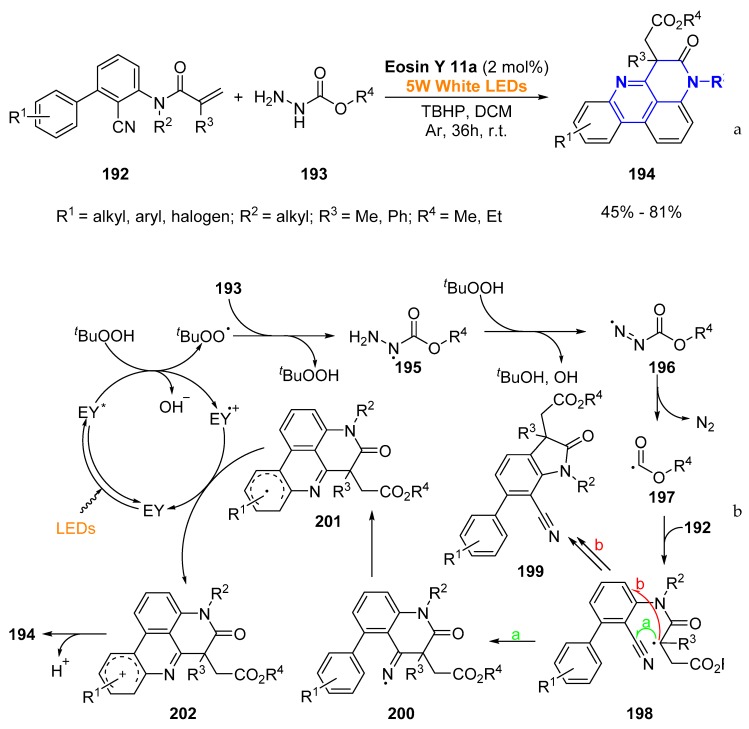
(**a**) Photoredox alkoxycarbonylation–addition–cyclization sequence; (**b**) Proposed mechanism.

The cyano moiety is widely used in organic synthesis because it undergoes many important transformations [85]. Sun et al. described alkoxycarbonylation–addition–cyclization sequence triggered by Eosin Y **11a** under white LEDs irradiation in the synthesis of polyheterocycles **194** [86]. This visible-light-induced cascade reaction is initiated by the intermolecular radical addition of alkyl carbazate **193** to a double bond of *N*-arylacrylamide **192** followed by cyano-mediated cyclization. Desired poly nitrogen containing heterocycles **194** can be prepared in up to 81% yield (Scheme 28a).

The suggested mechanism is depicted in Scheme 28b. Excited state of Eosin EY* catalyst formed upon white LEDs’ irradiation undergoes single electron transfer with TBHP producing *t*-butoxy radical, which detach hydrogen from carbazate **193** leading to the radical intermediate **195**. Sequential dehydrogenation of **195** followed by nitrogen release provides alkoxycarbonyl radical **197**. At this moment, generated radical **197** reacts with double bond of the starting material **192** leading to the intermediate **198**, which undergoes intramolecular cyclization with cyano group to the radical intermediate **200** (path a). Alternatively, the radical **198** can undergo intramolecular cyclization with the aromatic ring (path b) leading to the undesired product **199** in trace amount. The radical **200** undergoes another intramolecular cyclization producing conjugated radical **201**, which after oxidation by the Eosin Y cation radical (EY^•+^) forms intermediate **202**. Final product **194** is obtained by the deprotonation of intermediate **202**.

**Scheme 29:**
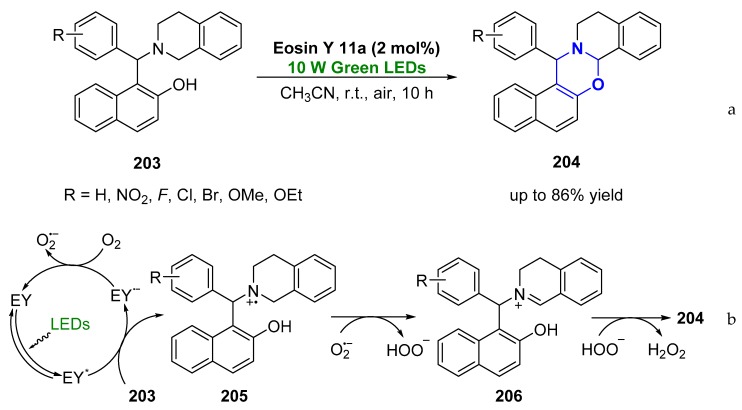
(**a**) Synthesis of 1,3-oxazines under photoredox conditions; (**b**) Proposed mechanism.

In the context of synthesis of THIQ analogues, Baruah et al. elaborated cross dehydrogenative approach toward 1,3-oxazines using visible-light-induced catalysis [87]. 1-Aminoalkyl-2-naphthols **203** easily undergoes intramolecular cyclization using Eosin Y **11a** as photoredox catalyst and green light source (Scheme 29a). On the basis of a series of control experiments, the authors proposed a plausible catalytic cycle (Scheme 29b). The excited Eosin Y (EY*) accepts an electron from the starting material **203** to form a cation radical **205**. Next, the generated EY^•−^ radical anion undergoes electron transfer with molecular oxygen regenerating the ground state Eosin Y **11a** and forming superoxide radical anion. Hydrogen removal from **205** and subsequent intramolecular addition of hydroxyl to the imine group provides the final product **204** in up to 86% yield.

**Scheme 30:**
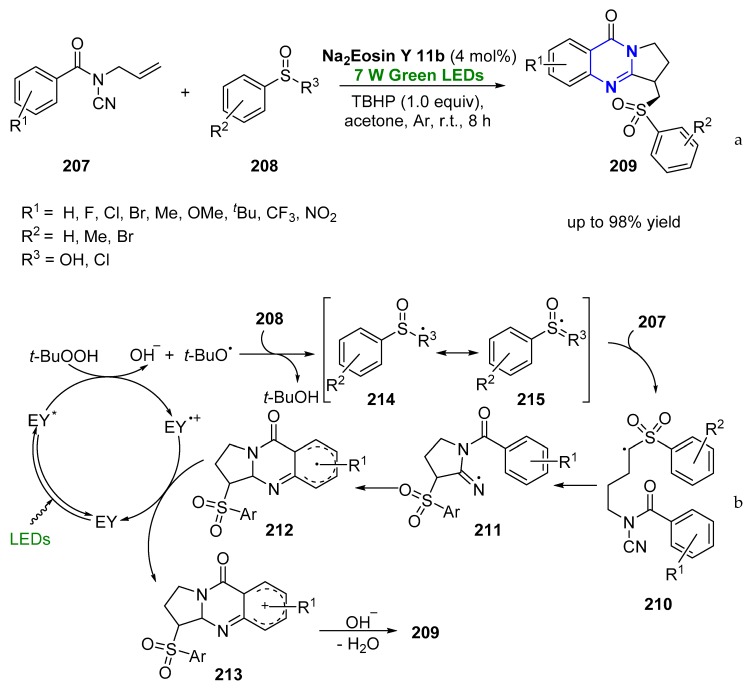
(**a**) Synthesis sulfonated quinazolinones via oxidative/reductive cyclization; (**b**) Proposed mechanism.

Quinazolinone structure is another important structural unit in heterocyclic chemistry [1,2,3]. Idrolone, Hydromox, and sildenafil citrate are the representative drugs containing quinazolinone motif. Pan and co-workers [88] demonstrated synthesis of sulfonated quinazolinones **209** in visible-light-induced oxidative/reductive cyclization of *N*-cyanamide alkenes **207** (Scheme 30a). Presented strategy features mild reaction conditions and broad substrate suitability. Various complex *N*-heterocycles **209** can be accessed in up to 98% yield. On the basis of their experimental observations and literature studies, authors proposed the mechanism depicted in Scheme 30b. Firstly, Eosin Y **11b** is irradiated by green LEDs to generate the excited-state Eosin Y (EY*). Secondly, electron transfer from Eosin to *tert*-butyl hydroperoxide (TBHP) generates *t*-butyloxy radical and OH^−^. Subsequent hydrogen atom removal from **214** produces sulfur-centered radical **215**, which undergoes addition to the double bond of **207** and produces radical intermediate **210**. Intramolecular cyclization cascade delivers radical **212**, which is oxidized by EY^•+^ to the cationic intermediate **213** through a single-electron-transfer route regenerating the same EY in its ground state. Finally, deprotonation of **213** gives the expected product **209**.

In addition to the given examples, other heterocycles containing multiple heteroatoms like imidazopyridines [89] or oxadiazoles [90] can be also obtained using metal-free visible-light-mediated catalysis.

## 3. Conclusions

In this review, recent advances in the synthesis of nitrogen and oxygen heterocyclic compounds via a metal-free visible-light-induced catalysis have been discussed. It has been shown that various metal-free organic dyes are effective photo-redox catalysts in the synthesis of many structurally different heterocycles containing one or more heteroatoms. Both non-aromatic and aromatic heterocyclic units are readily accessible by using visible-light-mediated catalysis in a straightforward modular way. Reported works indicate that organic dyes capable of visible-light-spectra absorption act as an attractive alternative to the transition-metal complexes. The variety of the presented examples indicates the potential use of the described methodologies in organic synthesis, drug discovery, or materials science. Moreover, the use of inexpensive organic, transition metal-free catalysts makes described protocols very practical and hold promise for broader application in industry and scientific community.
